# A qualitative study of safe abortion and post-abortion family planning service experiences of women attending private facilities in Kenya

**DOI:** 10.1186/s12978-018-0509-4

**Published:** 2018-04-24

**Authors:** Suzanne Penfold, Susy Wendot, Inviolata Nafula, Katharine Footman

**Affiliations:** 1 Marie Stopes International, 1 Conway Street, Fitzroy Square, London, W1T 6LP UK; 2 Marie Stopes Kenya, Kindaruma Road, P.O. Box 59328-00200, Nairobi, Kenya; 3 UCSF-Global programs, Morning Side Office Park, Ngong Road, P.O Box 40821-00100, Nairobi, Kenya

**Keywords:** Induced abortion, Kenya, Qualitative research, Private

## Abstract

**Background:**

To inform improvements in safe abortion and post-abortion family planning (PAFP) services, this study aimed to explore the pathways, decision-making, experiences and preferences of women receiving safe abortion and post-abortion family planning (PAFP) at private clinics in western Kenya.

**Methods:**

We conducted semi-structured interviews with 22 women who had recently used a safe abortion service from a private clinic. Interviews explored abortion-seeking behaviour and decision-making, abortion experience, use and knowledge of contraception, experience of PAFP counselling, and perceived facilitators of and challenges to family planning use.

**Results:**

Respondents discovered their pregnancies due to physical symptoms, which were confirmed using pregnancy testing kits, often purchased from pharmacies. Respondents usually discussed their abortion decision with their partner, and, sometimes, carefully-selected friends or family members. Some reported being referred to private clinics for abortion services directly from other providers. Others had more complex pathways, first seeking care from unsafe providers, trying to self-induce abortion, being turned away from alternative safe facilities that were closed or too busy, or taking time to gather financial resources to pay for care. Participants wanted to use abortion services at facilities reputed for being accessible, clean, medically safe, and offering quick, respectful, private and courteous services. Awareness of reputable clinics was gained through personal experience, and recommendations from contacts and other health providers.

Most participants had previously used contraception, with some reports of incorrect use and many reports of side effects. PAFP counselling was valued by clients, but some accounts suggested the counselling lacked comprehensive information. Many women chose contraception immediately following PAFP counselling; but others wanted to delay decision-making about contraception until the abortion was complete.

**Conclusion:**

Women’s pathways to safe abortion care can be complex, including use of multiple abortion methods, delays due to financial barriers, and challenges accessing safe providers. Improvements in community knowledge of safe abortion care and accessibility of services are needed to reduce recourse to unsafe abortion. PAFP counselling is valued by clients but quality of counselling can be improved by exploring women’s contraceptive histories, including information on more contraceptive methods, and inclusion of support for women who want to delay family planning uptake until their abortion is complete.

## Plain English summary

Legal restrictions on abortion were reduced in Kenya in 2010, but many women still use unsafe abortion methods. We interviewed 22 women who had recently used a safe abortion service from a private clinic about their pathways to care, how they chose the facility, their abortion and post-abortion contraceptive counselling experiences, and their use and knowledge of contraception.

Respondents discovered their pregnancies due to physical symptoms, which were confirmed using pregnancy testing kits, often purchased from pharmacies. Respondents usually discussed their abortion decision with their partner, and, sometimes, carefully-selected friends or family members. Some were referred directly to the private clinics for abortion services from other providers. Others had more complex pathways, first seeking care from unsafe providers, trying to self-induce abortion, attending alternative safe facilities that were closed or too busy, or taking time to gather finances to pay for care. Respondents sought safe, accessible, clean, respectful care from facilities that were viewed as reputable from personal experiences, and from friend, family and health provider recommendations. Respondents were often experienced contraceptive users but had discontinued due to side effects. PAFP counselling was valued by respondents, and many chose contraception immediately following counselling; others delayed contraceptive decision-making until after their recovery.

In conclusion, improvements in community knowledge of safe abortion care, accessibility of services, and PAFP counselling are needed to reduce recourse to unsafe abortion and prevent unintended pregnancy. Programmes that aim to reduce unsafe abortion should account for women’s complex pathways to care and influence their pathways away from unsafe methods and providers.

## Background

The World Health Organization (WHO) defines unsafe abortion as a procedure for terminating an unintended pregnancy carried out either by persons lacking the necessary skills or in an environment that does not conform to minimal medical standards, or both [1]. The persons with necessary skills and minimal medical standards are defined by the evolving WHO guidelines for safe abortion and health worker roles in safe abortion care [2–4]. In Kenya in 2010 the new constitution permitted abortion when, “in the opinion of a trained health professional, there is need for emergency treatment, or the life or health of the mother is in danger” [5]. However, the Kenyan penal code has not been updated to reflect changes in the Constitution, and the national *Standards and Guidelines on Reducing Maternal Mortality and Morbidity from Unsafe Abortion* were withdrawn in 2012 and have not been replaced [6]. The resulting confusion about the legal status of abortion restricts women’s access to safe services [7], and use of unsafe abortion providers and methods is still common [8].

In 2012, an estimated 119,112 women in Kenya received care for complications of unsafe abortion [9]. The reasons for use of unsafe services include the need for secrecy, uncertainty about the law, perceived higher cost of safe providers and lack of knowledge about abortion methods and safety [10, 11]. Increasing access to high-quality safe abortion care is crucial to prevent mortality and morbidity from unsafe abortion, which was responsible for about 13% of all maternal deaths globally in 2008 [12]. To prevent use of unsafe services, women’s pathways to abortion care must be better understood.

Among women who receive safe abortion services, increasing access to post-abortion family planning (PAFP) is also an important intervention to prevent subsequent unsafe abortion by preventing unintended pregnancies [12]. Contraception is widely available [13] and knowledge about contraception is high in Kenya, yet contraceptive use remains low [14]. A study in 2012 found that 49% of pregnancies in Kenya were unintended, 41% of which ended in abortion [15]. Another study in 2012 found that about 16% of women seeking post-abortion care following an unsafe abortion reported to have had a previous induced abortion [16]. Although evidence suggests that PAFP counselling can be effective in increasing women’s contraceptive uptake [17], inadequate counselling has been documented in private sector clinics in Kenya [18]. There is also limited evidence on the challenges that prevent women from using contraception after an abortion, and women’s preferences for PAFP counselling.

This study aimed to explore the decision-making, experiences and preferences of women who attended private clinics in Kenya for safe abortion services. The objectives of the study were (1) to understand women’s pathways to and experiences of seeking a safe abortion; (2) to understand the factors influencing the decision to seek a safe abortion service; (3) to describe women’s experiences of contraceptive use and PAFP counselling.

## Methods

We conducted this qualitative study in February 2016 as part of an evaluation of a quality management intervention to increase PAFP counselling and uptake in nine private clinics that are supported by the Marie Stopes Kenya ‘AMUA’ social franchise network in Western Region, Kenya [19]. During the pre-intervention phase of the evaluation, we interviewed women who had received an abortion or post-abortion care service at one of the nine clinics using a structured questionnaire on the day of procedure. During the informed consent process for this interview, we asked respondents whether they were interested in being contacted for a semi-structured interview at a later date. Respondents who were interested in taking part in an additional, longer interview were contacted by phone, up to 3 months after their abortion procedure.

We selected respondents for semi-structured interviews from six of the nine facilities, based on the number of respondents interested in taking part in an interview at each facility, and the geographic location of facility, to ensure that we selected both urban and rural clinics. We aimed to interview five respondents from each of the six facilities. We systematically selected interested respondents using a skip pattern, calculated based on the number of respondents expressing interest in participating from each facility, to ensure that respondents were selected from across the entire 3-month time period of the pre-intervention data collection.

Participant recruitment and data collection was conducted by six trained research assistants. The research assistants were all females aged 20–35 years, with Bachelor degrees and previous experience in qualitative interviewing. Research assistants received 2 days of training on conducting qualitative research, study procedures, informed consent, in-depth interviewing, self-reflection, the interview guide, and values clarification exercises. Practice interviews were used to ensure the research assistants could interact with clients in a sensitive, neutral and non-judgemental manner. Research assistants were supervised and supported by a field supervisor who listened to the recordings to assess the quality of the interviews and provided feedback to research assistants on points for improvement before their next interview.

We conducted the semi-structured interviews face-to-face in a private location convenient to the respondent, including clinics, hired rooms, and open spaces. We obtained individual written and oral informed consent to participate in the interview from each respondent at the start of each interview. Interviews were up to 1 h long and followed a detailed topic guide. The topic guide included questions about past and current experience and knowledge of contraception, experience of being counselled on and receiving contraception on the day of the procedure, perceptions of the barriers to family planning use, abortion-seeking behaviour, knowledge of abortion providers and experience at the clinic on the day of the abortion.

Interviews were conducted in a mixture of English, Swahili, and Luo depending on the location of the interview and the preferred language of the respondent. Interviews were audio-recorded and detailed field notes were made. Interviews were transcribed verbatim by the research assistants, and were then translated by a professional translator into English.

We conducted descriptive thematic analysis using both inductive and deductive coding, with the latter based on the research questions and topic guides [20, 21]. Data were coded in Microsoft Excel. Two coders (SP and KF) reviewed the first four interviews to reach consensus on coding structure.

## Results

### Facility and respondent characteristics

Twenty-two women participated in an interview. Respondents were recruited from three urban and three rural facilities in different counties of the western region of Kenya. The number of respondents per facility ranged from 2 to 5 (Table 1).

**Table 1:** Characteristics of Study Facilities

Facility	County	Urban / rural	Profession of main provider	Number of respondents (semi-structured interview)	Total number respondents interviewed during pre-intervention phase
1	Bungoma	Urban	Doctor	5	108
2	Uasin Gishu	Rural	Nurse	5	24
3	Trans-Nzoia	Urban	Clinical Officer	2	51
4	Migori	Rural	Nurse	2	8
5	Kisumu	Urban	Nurse	5	105
6	Homa Bay	Rural	Nurse	3	21

Respondents were most commonly in a relationship or married age 23–26 years and had children (Table 2). An equal number had been educated to primary level and college level (*n* = 9).

**Table 2:** Respondent Characteristics

Characteristic	Number (*N* = 22)
Marital status	
Married	8
With partner	10
Single	3
Divorced	1
Age (years)	
18–22	7
23–26	9
27–30	4
>30	2
Number of children	
0	8
1	6
2	2
>2	6
Employment	
Student	6
Unskilled	6
Skilled	5
Not working	5
Education level	
Primary	9
Secondary	4
College	9

### Pathways to seeking a safe abortion service

Respondents discovered their pregnancies due to physical symptoms such as weight gain, tiredness and nausea or vomiting in most cases, and more rarely due to a change in menstrual cycle. Just seven respondents mentioned a late period as being a sign of the pregnancy, while one realised her periods had stopped but did not associate this with pregnancy. Most respondents confirmed their pregnancy by using a pregnancy testing kit or service, often purchased from a pharmacy. Most respondents shared the news of the pregnancy with their partner or husband, and in some cases friends or family members. Information was shared selectively, with contacts that the respondents felt would support their decision or would not judge them. A few respondents told no one about the pregnancy or abortion, with one saying that *“I knew if I communicate even to one person, that information will spread that I have aborted”* (with partner, age 29, 1 child). Those who had shared their news were often advised on whether to terminate the pregnancy by their friends or family, and while some respondents were influenced by the opinions of partners or older family members, it was more common for the decision to be that of the respondent alone, sometimes going against advice of their partners as they felt *“it’s my choice to decide”* (Married, age 30, 2 children). The reasons for terminating the pregnancy were most commonly that respondents were still in education or were caring for another young child.

Some respondents reported being referred directly to the private clinics from other providers, including staff at dispensaries and chemists where they purchased the pregnancy test. However, in other cases, respondents’ pathways were longer and more complex. For example, a few respondents first sought abortion services through unsafe providers or tried to self-induce before accessing formal care. One respondent had tried traditional medicine (tea made from roots and bark) as a first abortion attempt, following advice from a cousin, and thought the tea had worked after her second attempt at using it (almost 2 weeks later): “*I was convinced that it has come out. But then I saw that I was feeling the way I used to feel, when it is there... …then I decided to go to the facility”* (Married, age 29, 5 children). One respondent reported receiving a heavy massage before seeking post-abortion care at the clinic, while two had tried medicines and either experienced complications or found the drugs were not effective. There were also reports of delays in accessing safe services because respondents sought abortion care from a formal provider first, but were not able to access the service because the facility was closed, could not be located, or was too busy. In one case, this resulted in a respondent obtaining pills from the chemist instead.

Some respondents also struggled to obtain money and had to delay obtaining the service while they gathered funds. However, most respondents viewed the services as good value:

*“What I had was little [money]... I left it with him and told him that I was going to look for the remaining... the next day I had got [the money] and came with it... It was not a lot and it was not little according to... how I wanted to get helped. I didn’t see it as expensive.”* (single, age 21, no children)

When recounting the abortion procedure itself at the facility, most respondents seemed to be well-counselled and informed about what would happen, including being given a choice of procedure, told what the procedure would involve, how long it would take, what level of pain and bleeding to expect and what to do in case of side effects. Respondents had varying information needs, with some wishing they had been given more information and others wanting to know very little about the abortion procedure out of fear of what it involved. Most respondents were satisfied with the clinic and the service they received and women commonly recalled feeling relieved after completing the abortion. However, in a few cases clients recalled a negative experience at certain clinics, being made to feel *“guilty”* because “*he said nothing but it was just actions, no advice, no talking...you just feel like you are doing the wrong thing...”* (with partner, age 21, no children).

### Factors influencing seeking a safe abortion

The most commonly-mentioned factors influencing respondents’ care-seeking were concerns of quality and safety of the service. Respondents had heard about others experiencing morbidity or mortality from unsafe abortion, and had knowledge of methods (traditional and herbal medicines, drinking tea or juice, inserting herbs into the vagina, and ‘pills’) which they viewed as unsafe. Fear of ineffective or unsafe methods and ‘fake’ providers was often a direct motivation for seeking formal abortion services:

*“It is better I go to a doctor where I will be safe, where even if bleed a lot, they will know how to help me. Now that is why I did not use any other methods because you hear someone else has gone and died here and actually they were trying to remove a pregnancy.” (married, age 26, 2 children)*

Prior experience of the clinic was an important influencing factor; either from the respondent previously using the facility for other services such as family planning, or from friends or family specifically recommending the facility. Perceptions of the safety of the procedure, cleanliness, competency and attitude of staff, speed of service and privacy; and the possibility of obtaining family planning afterwards were specific aspects of care that influenced the reputation of a clinic: *“she told me... that this place is very nice, things are done in a clean way and then I will be washed and I won’t have many complications. I told her then, take me there”* (with partner, age 20, no children).

As well as safety, respondents also valued discretion, both in terms of the provider and the method: *“I told her I don’t want pills for swallowing, I don’t want the one where I will bleed a lot... so that now my uncle, so that he knows”* (with partner, age 20, no children). The desire to be anonymous influenced the choice of clinic in some cases, with women deliberately travelling to a facility further away from home for this reason.

Ease of availability of abortion services was important, with some respondents mentioning they had not sought an abortion at public facilities because they feared being turned away. Cost of the service was also a consideration, with some facilities having a reputation for being more affordable. For some, cost was a low consideration relative to the need to get an abortion: *“I did not consider things to do with price. I just wanted it to be terminated”* (married, age 29, 5 children).

### PAFP and contraceptive experience

Most respondents were not using contraception at the time they conceived but some were using short-acting or traditional methods. Most respondents had experience of using several methods of contraception previously, most commonly injectables and condoms. There were many reports of side effects related to contraceptive use, which sometimes meant respondents stopped using contraception: *“Norplant was good for the first three months but onwards it started giving pressure in the chest, and I decided to terminate it”* (married, age 30, 2 children). Reasons for non-use of contraception included partner opposition and concerns about side effects. Respondents were knowledgeable about a range of contraceptives, and where to obtain them, with information commonly received from friends, family, school, health clinics, the internet, radio, television, printed media and church. However, misconceptions about some contraceptives were common.

Most respondents reported receiving some counselling about PAFP on the day of procedure. For a couple of respondents, contraceptive counselling was initiated by the respondent herself rather than the provider. Most respondents said that they were open to and appreciative of the counselling. Most commonly respondents remembered being counselled on the effectiveness of different contraceptive methods. In some cases, respondents reported being told about a wide range of contraceptive methods, but for others the options mentioned were limited to short-term methods such as condoms, oral contraceptive pills and injections. Some respondents remembered the providers explaining the benefits of contraception in terms of spacing pregnancy to enable women to care for young children they have and preventing future need for abortion. Some also spoke about being reassured about side effects of different methods, although more commonly respondents wished they had received more information about this. There were also a couple of cases where women felt they were just “being told to use family planning”. Three respondents reported that they received no counselling on PAFP but would have liked to have received more information, such as on how to prevent pregnancy and sexually transmitted infections, and how different contraceptive methods worked.

Of those respondents who did not take up contraception services on the day of the abortion, reasons included not being informed about contraception by the provider, being advised to seek contraception elsewhere or later, and fear of side effects resulting from their own experience or from the experiences of other people. Respondents also chose to delay contraceptive uptake because they planned to wait until after marriage, needed to discuss it with their partners to avoid “*chaos in [the] house*”, or wanted to complete the abortion first (owing to either the pain or anxiety of the abortion, or desire to know it was complete): *“I told him I will not…use for now; let me first finish that [abortion] medicine”* (with partner, age 23, no children). Some respondents reported obtaining contraception from elsewhere up to 1 month after the abortion, while others who intended to seek contraception after leaving the facility did not as they were too busy or forgot.

There was a range of reasons behind respondents’ decisions to use contraceptive services following an abortion, several of which reflected the content of the reported counselling they received. These included not wanting to have another abortion, concern about health effects of abortion and emergency contraception, having not previously used contraception and being informed for the first time by the provider, and realising the need for family planning as the respondent was young and still in school. The most common method used to prevent pregnancy post-abortion was the implant, but others included the oral contraceptive pill, injection, condoms and the intra-uterine device (IUD).

## Discussion

Our study found that several barriers and facilitators shaped respondents’ pathways to safe abortion care, including delays in discovering pregnancy and lack of recognition of pregnancy symptoms; use of pregnancy test kits; advice from partners and social networks; referrals from non-abortion providers; challenges accessing facilities; and financial constraints. Respondents seeking safe abortion services in private facilities were highly influenced by the reputation of safe medical care and fear of unsafe services, as the risks of unsafe abortion and commonly-used traditional methods were well-known. The clinics’ reputations of being affordable and discrete were also important. Respondents valued PAFP counselling, and, in some cases, counselling reportedly addressed specific barriers to contraceptive use, such as fear of side effects. However, counselling could be more targeted to not only address the effectiveness of methods, but also the benefits of family planning and how side effects can be managed. Most respondents had experience of using several contraceptive methods, and many reported that side effects had caused them to stop or change method, which is a common issue for continuation [22]. Several respondents wished that PAFP counselling had provided information on a wider range of contraception methods and their side effects. The range of methods available may have been hindered family planning use, as observed elsewhere [23]. Many respondents opted for contraception immediately post-abortion. However, some needed time to consider their options, discuss contraception with their partner, or complete the abortion process before making decisions about family planning.

This study had a number of strengths and limitations. To understand experiences of these sensitive topics, semi-structured interviews were the most appropriate technique. They were conducted by experienced, trained, female research assistants who were native speakers of the languages of the interviewees. However, it may be that some community aspects relating to abortion and contraception were missed, and these could have been better explored through focus group discussions. The interviews were conducted within a few months of respondents having their abortions, which reduced the chance of recall error of the abortion experience, but the timing of the interviews relative to the abortion varied between 1 and 3 months, so there was potential variation in the level of recall between respondents. This study explored the views and experiences of women who sought abortion services at private clinics in Kenya, and the results are not generalizable to women seeking abortions from other types of providers.

While respondents in our study viewed medical safety of the procedures to be an important influence on where they obtained their abortion, other evidence suggests that safety does not always have the same meaning or is not always a priority for care-seeking. A study of wider community views from western Kenya found that women felt that there was no such thing as a safe abortion method, and that cost and secrecy were more important than safety [11]. As in our study, respondents in a study by Izugbara et al. reported that dependable social networks were important in identifying an abortion provider, but this was viewed as an element of the safety of the procedure, more so than clinical safety [10]. Other elements of service safety reported by Izugbara et al. include confidentiality, concealment of the abortion, being shielded from the law, and cost [10]. A study in Zambia also found that for women seeking post-abortion care from hospitals, the need to conceal the abortion had outweighed concerns about safety [24]. Discretion of the abortion service was also prioritised by many respondents in our study, and lack of clarity about the legality of abortion and stigma associated with it may increase the value placed on discrete service provision [24].

The differences in care-seeking decisions that result in use of safe and unsafe services are likely to reflect differences in respondents’ backgrounds, such as access to money, levels of education, health knowledge, nature of social networks, and capacity to overcome challenges such as stigma and lack of accessible services. The study of community views in western Kenya found that the costs of safe providers prohibited their use [11]. Although this was not the case in our study, we note that we only interviewed women for whom finance was not an ultimate barrier to care. It is likely that these different views of cost may reflect higher levels of knowledge of the health system and socio-economic status among the respondents in our study. However, we also note that the socio-demographic characteristics of the respondents in the Izugbara et al. study do not appear to be substantially different from those in our study, suggesting that there may also be an element of luck in what advice is received and by whom at the exact moment a woman is seeking an abortion, particularly in the context where there is a lack of accurate information available, and there are large numbers of options available, many of which are unsafe.

The study findings highlight potential opportunities for programmes to influence women’s abortion seeking pathways to reduce recourse to unsafe abortion. Abortion pathways were complex in some cases, with multiple options being tried or used before finally accessing a safe service, as previously noted in other studies [25, 26]. Improving awareness of pregnancy symptoms, such as missed periods, may reduce delays in care-seeking. Ensuring pregnancy test kits are widely available and have links to a source of support for pregnancy crisis counselling, for example through a hotline or pharmacy referrals to clinic-based care, may increase awareness of safe abortion methods at earlier gestational ages [27]. Referrals facilitated respondents’ access to safe services in some cases, and strengthening referral networks from a range of non-abortion and informal providers could also help to reduce delays in care-seeking and promote safe service use. Social networks played a key role in respondents’ pathways, with clinic recommendations often coming from friends or family. Husbands and partners were most commonly confided in about the need for abortion, so increasing men’s awareness of the risks of unsafe abortion and the availability of safe options may also influence women’s awareness and use of safe methods. In some cases, respondents attempted to access safe services but found that clinics were not open or were too busy, so there is a need to increase the availability of safe services. As recommendations from friends influence the use of safe options, care must be woman-centred and de-stigmatising to ensure that use of safe options is replicated through word of mouth. Finally, the content of PAFP counselling should take women’s contraceptive preferences and previous experience into account, include information on a wide range of methods, reassure women on how to deal with side effects, and address common misconceptions [28]. PAFP counselling should acknowledge that women may not be ready to make a decision on the day of the abortion procedure, and find ways to support women after they leave the clinic, either through mobile technologies or in-person support.

## Conclusion and recommendations

Unsafe abortion is common in Kenya. Our study of the pathways and experiences of women using safe providers in western Kenya highlights the opportunities to reduce use of unsafe options and ensure women access safe options. These include reducing delays in discovery of pregnancy through improved education on pregnancy symptoms; ensuring pregnancy test kits have referral links to pregnancy crisis counselling; improving community knowledge of abortion safety and options due to the important role of advice from social networks, and particularly men due to the important role of husbands and partners; building referral networks from non-abortion and informal providers to improving referrals to safe providers; and improving the accessibility and availability of safe providers.

PAFP counselling is valued by clients and can improve access to information and clarify misconceptions, but improvements in the quality and content of counselling may further facilitate contraceptive use. While PAFP counselling should prioritise method provision immediately after an abortion to maximise protection and reduce incidental costs for repeat facility visits, it should also include steps to follow-up and support women who want to use family planning at a later date.

## Abbreviations

IDI: In-depth interview
IUD: Intra-uterine device
KEMRI: Kenya Medical Research Institute
MSI: Marie Stopes International
PAFP: Post-abortion family planning
